# Studies on the binding affinity of anticancer drug mitoxantrone to chromatin, DNA and histone proteins

**DOI:** 10.1186/1423-0127-16-31

**Published:** 2009-03-11

**Authors:** Zahra Hajihassan, Azra Rabbani-Chadegani

**Affiliations:** 1 Department of Biochemistry, Institute of Biochemistry and Biophysics, University of Tehran, Tehran, Iran

## Abstract

Mitoxantrone is a potent antitumor drug, widely used in the treatment of various cancers. In the present study, we have investigated and compared the affinity of anticancer drug, mitoxantrone, to EDTA-soluble chromatin (SE-chromatin), DNA and histones employing UV/Vis, fluorescence, CD spectroscopy, gel electrophoresis and equilibrium dialysis techniques. The results showed that the interaction of mitoxantrone with SE-chromatin proceeds into compaction/aggregation as revealed by reduction in the absorbencies at 608 and 260 nm (hypochromicity) and disappearance of both histones and DNA on the gels. Mitoxantrone interacts strongly with histone proteins in solution making structural changes in the molecule as shown by CD and fluorescence analysis. The binding isotherms demonstrate a positive cooperative binding pattern for the chromatin- mitoxantrone interaction. It is suggested higher binding affinity of mitoxantrone to chromatin compared to DNA implying that the histone proteins may play an important role in the chromatin- mitoxantrone interaction process.

## 

Mitoxantrone is a synthetic antineoplastic drug, structurally similar to the anthracyclines, widely used as a potent chemotherapeutic agent in the treatment of various cancers such as advanced breast cancer, lymphoma and leukemia [1-3]. Widespread interest in mitoxantrone has arisen because of its apparent lower risk of cardiotoxic effects compared with the naturally occurring anthracyclines [4,5].

Numerous studies on the mechanism of mitoxantrone action indicate that nuclear DNA is the major target for this drug [6,7]. The structure of mitoxantrone lacks the amino sugar moiety and tetracyclic ring (A) of anthracyclines but has a planar anthraquinone ring which intercalates between DNA base pairs and the nitrogen-containing side chains bind the negatively charged phosphate groups of DNA [7,8]. Binding of mitoxantrone to DNA causes DNA condensation and inhibits DNA replication and RNA transcription [9-11]. Also it is a potent inhibitor of topoisomeraseII, an enzyme known to be important for the repair of damaged DNA and this leads to single and double strand breaks [12].

The binding of mitoxantrone to DNA has been studied in detail [13-15]. However, in the cell nucleus; DNA is compacted into a complex structure built from the interaction of histones with DNA named nucleosomes. These consist of 145 base pairs DNA wrapped around an octamer of core histones. There are 5 main histones: the linker histones of the H1 family and 4 core histones (H2A, H2B, H3 and H4) which are arranged in an octamer form [16]. How the presence of chromosomal proteins affect and modulate the binding of intercalating drugs to DNA, is an important question when trying to understand the mechanism of drug action at the chromatin level. To explore this question, we employed several experiments designed to clarify this question in more detail.

In the present study, we attempted to examine and compare the effect of mitoxantrone on rat liver chromatin, DNA and histone proteins in solution to further understand the mechanism of drug action at the cellular chromatin level. The results provide evidence that mitoxantrone shows different affinity to chromatin, DNA and histones implying that the environment of chromatin plays fundamental role in the drug-DNA interactions.

## Materials and methods

### Materials

Mitoxantrone hydrochloride (2 mg/ml in H_2_O) was purchased from Helale Ahmar, Tehran, Iran (manufactured by Ebewe Pharma Ges.m.b. Austria) stored at 4°C in the dark. Before use, it was diluted to desired concentrations with 10 mM Tris-HCl (pH 7.4) and its concentration was determined spectrophotometrically using a molar extinction coefficient of 19 200 M^-1 ^cm^-1 ^at 608 nm. Microccocal nuclease (MNase), proteinase K, ECoR1-Hind III digested DNA marker, cocktail protease inhibitor were from Sigma Chemical Company. Calf thymus DNA (Sigma) was dissolved in 10 mM Tris-HCl (pH 7.4), dialyzed overnight against the same buffer and its concentration was determined using an extinction coefficient of 20 cm^-1 ^mg^-1 ^at 260 nm. Albino rats weighting of 150–200 gram of either sex were used throughout the experiments.

### Preparation of chromatin, DNA and histones

Nuclei were prepared from rat liver as described elsewhere [17]. All steps were carried out at 4°C in the presence of protease inhibitor PMSF at a final concentration of 1 mM or cocktail protease inhibitor (1/100 V/V). EDTA soluble chromatin was isolated according to the procedure reported before [18] with some modifications. Briefly, the purified nuclei were suspended in digestion buffer (0.25 M sucrose, 25 mM NaCl, 1 mM CaCl2 and 10 mM Tris-HCl (pH 7.4)) and DNA content was determined by measuring the absorbance at 260 nm. The nuclear suspension at A_260 _= 100 was digested with 3 units of microccoccal nuclease/mg of DNA at 37°C for 10 min. The solution was brought to 10 mM EDTA on ice, centrifuged at 8000 g for 5 min and then the chromatin solubilized in 0.25 mM EDTA (pH 8). The chromatin thus prepared designated as EDTA-soluble chromatin (SE-chromatin).

DNA was isolated from the SE-chromatin samples by proteinase K digestion (1 μg of enzyme/10 μg of DNA) and phenol-chloroform extraction method. The extracted DNA was then resuspended in 10 mM Tris-HCl (pH 7.4). The concentration of DNA in both, the SE-chromatin and purified DNA, were determined spectrophotometrically using an extinction coefficient of 20 cm^-1 ^mg^-1 ^at 260 nm.

Whole histone, consisting of all five histones, was extracted from calf thymus by 0.3 N HCl as described by Johns [19] and further purified using DEAE ion exchange chromatography. The whole histone was dissolved in 10 mM Tris-HCl (pH 7.3) and after pH adjustment; the solution was stored at -20°C and used within a month.

### UV/Vis Spectroscopy

The SE-chromatin, DNA and histones [100 μg/ml DNA or histone in 10 mM Tris-HCl (pH 7.4)] were incubated individually with appropriate concentrations of mitoxantrone for 45 min at room temperature in the dark. Control samples containing equal volumes of SE chromatin, DNA and histones in the same buffer were incubated along with the drug treated samples under the same condition.

Mitoxantrone treated and the controls were subjected to spectroscopic analysis using Shimadzo UV-160 spectrophotometer, equipped with quartz cuvettes. The wavelength of 400 nm was selected to detect the amount of turbidity. After centrifugation at 8000 g for 1 min, absorbance of the supernatants was recorded using multi λ system.

### Fluorescence Spectroscopy

The measurements were performed on a Carry Eclipse fluorescence spectrophotometer equipped with a thermostatically controlled cell holder at ambient temperature. The monochromatic slits were set at 5 nm to reduce the intensity of the signal depending on the experiment. All samples were made in 10 mM Tris-HCl (pH 7.3) at 20°C and quartz fluorescence cell of 1 cm path length was used. Protein solution (5–10 μM) was titrated with aliquots of mitoxantrone and equilibrated until a steady emission reading was obtained. The accumulated volume of titration was less than 10 μl, so dilution effect was negligible. The spectra were recorded between 290–410 nm after excitation at 278 nm. Mitoxantrone, histones and amino acid tyrosine were prepared individually in the same buffer and their emission spectra recorded in the same condition and used as a control.

### Circular dichroism experiment

Circular dichroism (CD) experiment was performed using CD spectrometer model 215 (AVIV instruments INC.). The Far-UV CD spectra of histones in 10 mM Tris-HCl pH 7.3 and in the absence and presence of various concentrations of mitoxantrone was recorded in the range of 190–260 nm with a spectral resolution of 1 nm. The scan speed was 20 nm/min and the response time was 0.3330 sec with a band width of 1 nm. Quartz cell with a path length of 10 mm was used and all measurements were carried out at 25°C. Results are expressed as molar ellipticity expressed as [*θ*], in deg × cm^2 ^× dmol^-1^.

### Gel electrophoresis

The supernatants obtained from the interaction of SE-chromatin with various concentrations of mitoxantrone were concentrated using Eppendorf concentrator (model 5301) and then analyzed on 15% SDS polyacrylamid gel electrophoresis as described by Laemmli [20]. The gel was stained with coomassie brilliant blue R 250, destained in methanol/acetic acid and photographed.

Agarose (1.5%) gel electrophoresis was also carried out in TBE buffer [89 mM Tris-borate, 2 mM EDTA (pH 8)] at a constant voltage of 80 V for 1 h [21]. The gel was then stained with ethidium bromide (5 μg/ml in distilled water) and photographed.

### Equilibrium dialysis

SE-chromatin and DNA were prepared in 10 mM Tris-HCl (pH 7.4) and dialyzed against the same buffer containing serial concentrations of mitoxantrone using Spectrum Laboratories (USA) dialysis tubing at room temperature. The equilibrium was achieved within 72 h at 23°C. The total drug concentration (C_t_) and the concentration of free drug (C_f_) in the dialysate were measured directly from the absorbance at 608 nm before and after dialysis using extinction coefficient of 19 200 M^-1 ^cm^-1^. The amount of bound drug (C_b_) was obtained from C_b _= C_t_-C_f_. Binding parameters were determined from the plot of r/C_f _versus r according to Scatchard method [22], where r is the ratio of bound drug to total base pair concentration and C_f _is the amount of free drug. In this presentation, n (the apparent number of binding sites), is the intercept of the linear region of the binding curve with the horizontal axis. Also K (apparent binding constant) corresponds to the negative value of the slope of the curve. The Hill coefficient value (n_H_) was determined from the slope of the ln (r/n-r) versus ln C_f _according to the Hill equation [23].

## Results

### Mitoxantrone shows higher affinity to chromatin compared to DNA

**Figure 1:**
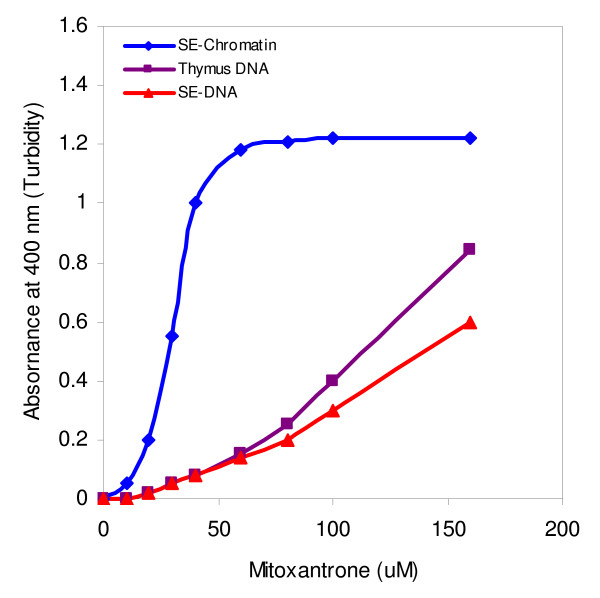
**Turbidity measurements at 400 nm of the interaction of various concentrations of mitoxantrone with SE-chromatin (diamond), SE-DNA (triangle) and thymus DNA (square)**. The reaction was carried out in 10 mM Tris-HCl (pH 7.5) and incubation time was 45 min at 23°C. Results are average of 3 individual experiments.

An experiment was designed using rat liver soluble chromatin fraction incubated in the presence and absence of the various concentrations of mitoxantrone. Addition of drug to SE-chromatin solution resulted in chromatin aggregation and precipitation which could be detected by monitoring the absorbance at 400 nm (turbidity). The result is shown in Fig. 1. As is seen, gradual increase in mitoxantrone concentration enhances turbidity of the reaction mixture. Turbidity formation is slow at low concentrations of drug (up to 20 μM), but a sharp absorbance changes is occurred upon increasing drug concentration (between 20 and 40 μM).

To compare data with the DNA free in solution (in the absence of chromatin proteins), DNA was isolated from such SE-chromatin as described in the method section (designated SE-DNA) and treated with various concentrations of mitoxantrone in the same experimental conditions mentioned for the SE-chromatin. Calf thymus DNA was also used for comparison. The results obtained from turbidity measurement are inserted in Fig. 1. The absorbance changes of both SE-DNA and thymus DNA in the presence of mitoxantrone exhibited similar but not identical binding pattern which differs considerably compared to SE-chromatin- mitoxantrone complex pattern. In the case of both DNAs used, the absorbance changes at 400 nm is very slow up to 80 μM of drug and then changes slightly, thus much higher drug concentration is needed to obtain saturation state.

**Figure 2:**
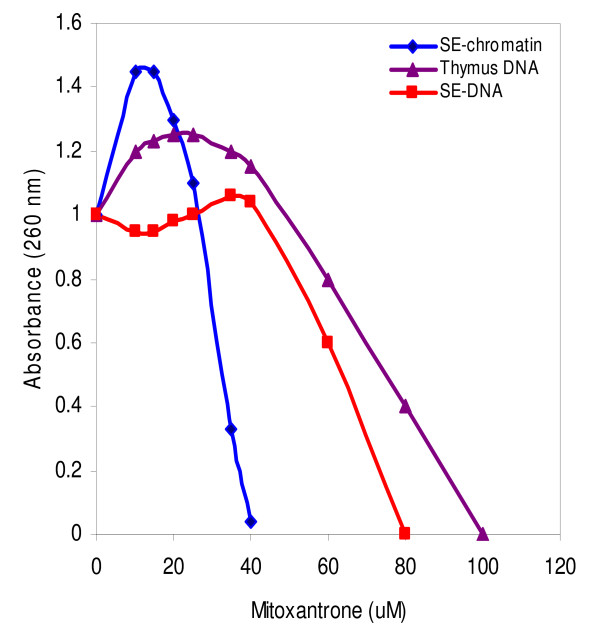
**Absorbance changes at 260 nm of the supernatants obtained from the interaction of mitoxantrone with SE-chromatin (diamond), SE-DNA (triangle) and calf thymus DNA (square)**. Experimental condition was the same as described in figure 1.

In the next step, the precipitates were removed by brief centrifugation and the clear supernatants were subjected to UV/Vis spectroscopy measurements. The result of absorbance changes at 260 nm is shown in Fig. 2. For the SE-chromatin, and at low concentrations of drug (≤ 10 μM), a slight increase in the absorbance is observed, but at higher drug values the absorbance is considerably decreased and drops to zero at 40 μM of mitoxantrone. Whereas in the case of thymus DNA and SE-DNA, the absorbance changes at 260 nm occurs beyond this range (80 and 100 μM of mitoxantrone respectively).

### Mitoxantrone binds to histones and DNA as analyzed on gel electrophoresis

**Figure 3:**
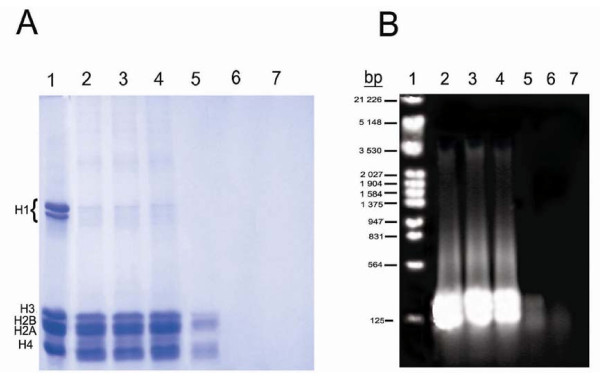
**A: 15% SDS gel electrophoresis of the supernatants obtained from the interaction of various concentrations of mitoxantrone with SE-chromatin**. Lanes 2–7 are 0, 10, 20, 30, 40 and 60 μM of mitoxantrone respectively. Lane 1, calf thymus histones as a standard. **B**: Agarose gel of DNA extracted from the supernatants. Lane 1, EcoR1 DNA marker. Lanes 2–7 are the same as in gel A. Number of experiments was 3.

In order to obtain information about the possible effect of mitoxantrone on SE-chromatin components, the supernatants obtained from the interaction of mitoxantrone with the chromatin were analyzed on agarose and SDS polyacrylamide gel electrophoresis (Fig. 3). On the SDS gel (Fig 3A), in the absence of mitoxantrone, the proteins released from the chromatin into solution are mostly core histones and a trace of histone H1 (lane 2) as analyzed parallel to the thymus histones (lane 1). This spontaneous release of proteins has also been observed by Bartkowiak [24] and represents the background of the experiment. In the presence of mitoxantrone and at low concentrations (<20 μg/ml), the proteins pattern is very similar to the control but at higher concentrations (>20 μg/ml), induce chromatin compaction and tends the histones to be un-extractable from the mitoxantrone treated samples leading to their disappearance on the gel (Fig. 3A lanes 5–7).

Moreover, electrophoresis of DNA extracted from the mitoxantrone treated samples and the control, shown in Fig 3B, demonstrates that gradual increase in drug concentration removes more DNA from the reaction mixture.

### Cooperative binding of mitoxantrone to chromatin

**Figure 4:**
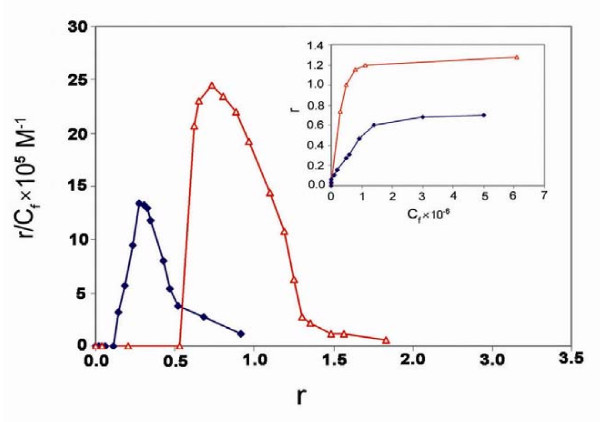
**Scatchard plots of the binding of mitoxantrone to SE-chromatin (diamond) and DNA (triangle) carried out in 10 mM Tris-HCl pH 7.4 for 72 hours at 25°C**. Results are means of 3 individual experiments.

The binding isotherm obtained from the equilibrium dialysis experiment is shown in Fig. 4. The Scatchard plot of the binding of mitoxantrone to SE-chromatin exhibits a cooperative binding behavior, as illustrated by the positive slope observed in the low r regions of the binding isotherm. The curve reaches a maximum at a value of r = 0.27 and decrease in the slope is observed at higher r values. Drawing r versus C_f_, as shown in the insert of Fig 4, clearly demonstrates a sigmoid curve, that is, as C_f _increases, r rises rapidly and then levels off, indicating that the system approaches to equilibrium or saturation. The binding of mitoxantrone to chromatin represents a binding constant of k = 7.1 × 10^6 ^M^-1 ^and drawing ln r/n-r against ln C_f _gives a straight line with a slope of the n_H _(Hill coefficient) which was 3, confirming the positive cooperative binding of drug to chromatin.

The binding isotherm of DNA is also given in Fig. 4. It also exhibits cooperative binding pattern with a binding constant of k = 5.0 × 10^6 ^M^-1 ^and n_H _value of 1.9. The result clearly demonstrates lower binding affinity of mitoxantrone to DNA compared to chromatin. The result is in agreement with the cooperative binding of mitoxantrone to DNA reported previously [25].

### Mitoxantrone also interacts with the histones free in solution

**Figure 5:**
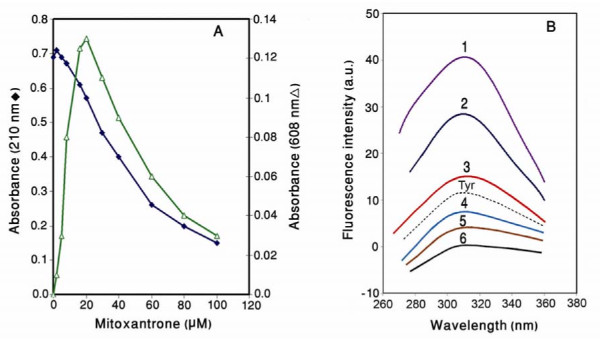
**A) Absorbance changes of histones upon mitoxantrone binding**. The reaction was carried out in 10 mM Tris-HCl pH 7.3 at 23°C. B) Fluorescence emission spectra of the histones in the presence and absence of various concentrations of mitoxantrone. All samples were prepared in 10 mM Tris – HCl (pH 7.3) and incubation time after drug addition was 20 minutes. Excitation was at 278 nm. Spectra 1–6 are 0, 10, 20, 30, 40, and 60 μM of mitoxantrone respectively. Tyr: tyrosine spectrum given for comparison.

Absorbance in the UV/Vis region has been successfully used for the analysis of antracyclines-linker histones interactions [26,27]. In this study, the purified histone proteins were incubated in the presence and absence of various concentrations of mitoxantrone and then absorbance changes were monitored at 608 and 210 nm. Figure 5A shows the absorbance changes of the histones as a function of drug concentration. As is seen, the absorbance at 210 nm is considerably decreased upon addition of mitoxantrone. This absorbance reduction is also observed in the pattern of 480 nm which is exclusively related to the wavelength of mitoxantrone. The results indicate that drug molecules inter the sites of histones, which are active in electron excitation.

The fluorescent emission spectra obtained from the interaction of mitoxantrone with the histones is also shown in Fig 5B. Histones, in the absence of mitoxantrone exhibit emission spectra in the position corresponding to tyrosine emission with a maximum intensity at 305 nm (fluorescence emission spectra of tyrosine has also been provided for comparison). Addition of mitoxantrone to the histone solution reduces the fluorescence intensity of the histones without any red shift in the emission maxima (I_max_) as drug concentration is increased. This clearly implies that the binding of mitoxantrone to histones is dependent on their accessibility to the environment.

**Figure 6:**
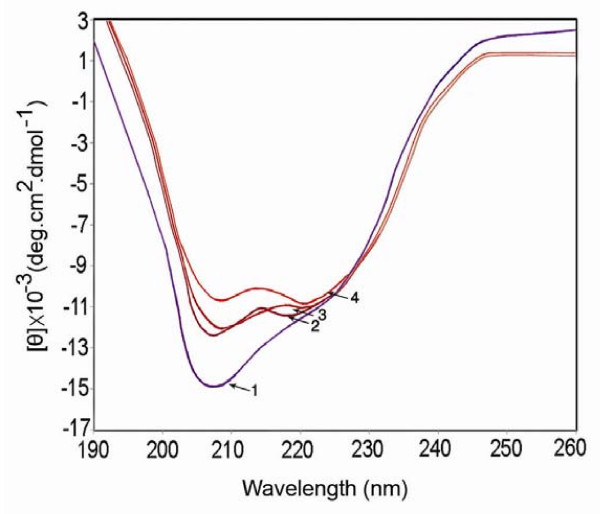
**Far UV CD spectra of the histones in 10 mM Tris-HCl at pH 7.3 in the absence and presence of mitoxantrone**. Each spectrum was obtained at 25°C with a 10 mm path length cell. 1–4 are 0, 10, 20 and 40 μM of mitoxantrone respectively. All necessary corrections were made for background absorption.

To obtain further information about the binding of mitoxantrone to histone proteins, we compared the secondary structure of the intact and histone- mitoxantrone complex in the same experimental condition. As is shown in Fig. 6, histones display a CD spectrum with negative extremes at 209 and 220 nm (the former more intense); whereas, mitoxantrone treatment caused a significant decrease in the intensity of the peaks, especially at 209 nm, which implies that, the secondary structure of the protein is perturbed upon drug binding.

## Discussion

Mitoxantrone is an anthracycline analog with potent antineoplastic activity and is distinctly different from doxorubicin in its cytotoxicity to leukemia cells [28]. Numerous studies on the binding of mitoxantrone to DNA have been undertaken and all indicate that it exerts its biological function by intercalation and also electrostatic cross-links within DNA to stabilize the binding [7,8]. In the cell nucleus, DNA is complexed with the histone proteins making a defined structure known as nucleosome [16], the structure that has been considered as a tool for the study of genome function in cancer [29]. Therefore the interaction of mitoxantrone with this nucleoprotein complex and its individual components, will clarify the real mechanism of drug action in the cell nucleus.

Although several attempts have been made to understand the mechanism of mitoxantrone action [13,14,30], the environment of nucleic acids in the cell nucleus, especially DNA-protein complex, may significantly modulate the interaction. Despite the presence of numerous published papers on the interaction of mitoxantrone with DNA, to date, the effect of mitoxantrone on chromatin has not been fully understood. In fact, this is the first paper demonstrating the effect of mitoxantrone on chromatin and histones protein (in the absence of DNA) in solution.

Our results suggest that mitoxantrone recognizes the chromatin structure with higher affinity than free DNA. Slight increase in absorbance at 260 nm announces induction of chromatin unfolding at very low concentration of mitoxantrone (<10 μM), however, at higher concentrations the interaction of mitoxantrone with chromatin is accompanied by chromatin compaction/aggregation. The binding of mitoxantrone to chromatin produces a very compact structure that prevents the release or extraction of histone proteins from such drug treated chromatin. Higher affinity of mitoxantrone to chromatin is also confirmed by the results obtained from the binding isotherms. Although the binding is positive cooperative for both DNA and chromatin, but K values are different. Moreover, mitoxantrone strongly binds to histone proteins in solution, and upon drug action, their secondary structure is reduced. Fluorescence emission spectra exhibits quenching of mitoxantrone with tyrosine chromophores of the histones which are mainly located in their globular domain. Severe binding of mitoxantrone to histones implies that apart from DNA, histone proteins are also a good candidate for mitoxantrone action at the chromatin level; however, the mode of interaction is still unknown.

From the results it also seems that the behavior of mitoxantrone -chromatin interaction differs considerably from the interaction of anthracycline anticancer drugs such as daunomycin and Adriamycin with chromatin. The anthracyclines preferentially bind to less condensed structure of chromatin and the order is DNA>chromatin-H1>chromatin [31,18], whereas in the case of mitoxantrone, the affinity of drug to chromatin is higher than to DNA.

In cancer cells, double stranded DNA is also associated with histone proteins making nucleosomes, but because of genetic or epigenetic changes of chromatin, the structure is more relaxed and loose compared to normal cells [32]. This implies that, in cancer cells, mitoxantrone also preferentially binds to nucleosome structure or partially histone free DNA rather than DNA completely free of histone (naked DNA). This is confirmed by our founding that mitoxantrone shows strong affinity to histone proteins. Although Oliveira Brett et al. (1998) have suggested preferential interaction of mitoxantrone with single stranded DNA using acidic condition [33]; we can not speculate this phenomenon which needs extensive work to be clarified.

Taking all together, it is finally concluded that mitoxantrone binds to chromatin and produces a compact structure, the statement which is in good agreement with the anticancer activity of the drug which inhibits DNA and RNA synthesis [10,11]. The results come in support of the notion that the protein component of chromatin can also be considered as a target for the activity of this antitumor drug. Whether mitoxantrone interacts directly with the histone proteins or cross linking occurs between the DNA and histones is still unknown. Further insights into the mechanism of mitoxantrone action at the chromatin level especially the real binding sites on histone proteins remain to be elucidated.

## Abbreviations

MNase: micrococcal nuclease; EDTA: ethylenediaminotetraacetic acid; PMSF: phenylmethylsulphonylfloride; SDS: sodium dodecyl sulphate; SE-Chromatin: EDTA Soluble Chromatin.

## Competing interests

The authors declare that they have no competing interests.

## Authors' contributions

Authors AR (as a PhD student supervisor) and ZH (as a PhD student), both have made substantial contributions to conceptions of design, acquisition, analysis and interpretation of data. Also the authors have been involved in drafting the manuscript, revising and approval of the final version to be published.
